# Professional Feeding Guidance Improved Infants’ Self-Feeding Proportion during Complementary Foods Introduction in Beijing, China: An Exploratory Study

**DOI:** 10.3390/children10111740

**Published:** 2023-10-26

**Authors:** Guochao Song, Jiahui Chang, Hongyan Guan, Yanfeng Zhang, Ting Zhang, Zhaofeng Zhang

**Affiliations:** 1 Capital Institute of Pediatrics-Peking University Teaching Hospital, Beijing 100020, China; gloriasong@stu.pku.edu.cn; 2 Beijing Municipal Key Laboratory of Child Development and Nutriomics, Beijing 100020, China; j.chang1220@student.pumc.edu.cn (J.C.); hongyanguan@126.com (H.G.); 3 Children’s Hospital Capital Institute of Pediatrics, Chinese Academy of Medical Sciences & Peking Union Medical College, Beijing 100020, China; 4 Nurturing Care Research and Guidance Center, Capital Institute of Pediatrics, Beijing 100020, China; 5 Department of Integrated Early Childhood Development, Capital Institute of Pediatrics, Beijing 100020, China; summyzh@126.com; 6 Department of Nutrition and Food Hygiene, School of Public Health, Peking University, Beijing 100191, China; 7 Beijing’s Key Laboratory of Food Safety Toxicology Research and Evaluation, Beijing 100191, China

**Keywords:** infants, complementary foods, feeding patterns, self-feeding, feeding guidance

## Abstract

An exploratory study was undertaken to examine the prevalence of infants’ feeding patterns in Beijing, China, as well as the factors linked to infants’ self-feeding proportion during the introduction of complementary foods, and the impact of professional feeding guidance on this proportion. A total of 122 families with infants aged 6–11 months from Beijing were included in the study. A descriptive analysis was employed to assess the prevalence of infants’ feeding patterns, while generalized linear model analysis was utilized to investigate the factors associated with these patterns. All families were provided with comprehensive and personalized professional guidance regarding the introduction of complementary foods for infants. However, 64 families were lost to follow-up, leaving 58 families who were re-evaluated and queried after one month. To exclude the influence of infants aging, both the 64 families prior to receiving feeding guidance, and the 58 families after receiving feeding guidance, were included in the analysis. The families with infants aged 6–8 months and 9–11 months were compared separately based on the presence or absence of feeding guidance. Statistical tests, including the Wilcoxon rank-sum test and χ^2^ test, were conducted to assess any significant differences. The study revealed that the proportion of infants engaging in self-feeding was found to be remarkably low (10% [0%, 40%]). Furthermore, a significant positive association was observed between the proportion of infants engaging in self-feeding and their age (*p* < 0.001). Notably, after receiving professional feeding guidance, the proportion of infants engaging in self-feeding significantly increased (from 1% [0%, 20%] to 30% [10%, 50%], *p* < 0.001 for infants aged 6–8 months; from 20% [10%, 50%] to 40% [30%, 50%], *p* < 0.001 for infants aged 9–11 months). These findings contribute valuable insights for improving postnatal care practices during the introduction of complementary foods for infants.

## 1. Introduction

A nurturing environment is especially important for a child’s whole life. Within the context of their nutritional development, the introduction of complementary foods during infancy represents a pivotal milestone. The WHO recommends that infants commence the consumption of complementary foods at the age of 6 months, alongside continued breastfeeding [1]. Various approaches exist for introducing complementary foods to infants, which can be categorized into two distinct groups based on the primary target audience: Baby-Led Weaning (BLW), wherein infants predominantly engage in self-feeding, accounting for over 50% of their feeding time, and Parent-Led Weaning (PLW), wherein infants engage in self-feeding for less than 50% of their feeding time. The feeding patterns have a noteworthy influence on infants’ development.

Aligning infant feeding practices with their individual needs, taking into account the development of appetite regulation and granting infants autonomy in determining their intake, is advantageous [2]. Baby-led weaning (BLW), an approach to complementary feeding that encourages infant independence by introducing finger foods instead of pureed food, has been found to be enjoyable for caregivers and promotes independent eating, as observed in studies conducted by Brown and Lee [3,4,5]. Furthermore, BLW has been linked to a potential decrease in food aversions among infants [6]. Advocates of the BLW approach contend that it facilitates the development of more nutritious dietary preferences in infants, as they are exposed to a broader range of food options and have the opportunity to observe the eating habits of their family members, thereby receiving positive role modeling [7,8,9].

In addition to the nutritional benefits, independent eating in infants presents various benefits that contribute to their overall development. The proportion of BLW was found to have a positive correlation with achieving the milestone of sitting unsupported at an earlier stage [10], while a reduced reliance on spoon-feeding by caregivers was associated with an earlier onset of crawling [10,11]. Furthermore, independent eating has been linked to enhanced motor development, fine motor skills, coordination [12], as well as language production and comprehension [11] in infants. Rapley’s research [13] supports the notion that independent eating provides an opportunity for infants to enhance their sensory development.

Furthermore, the WHO recommends that the introduction of “finger foods” (independent snacks for children) can commence at 8 months for the majority of infants [1]. It is noteworthy that many infants begin to exhibit the capability to grasp and consume finger foods between the ages of 4 and 7 months [14], indicating a mature ability for a significant number of infants. Nevertheless, it is important to acknowledge that self-feeding is not yet a customary aspect of their meals by the age of 8 months. Consequently, BLW is not as widespread as initially perceived and exhibits variations among countries [15]. For instance, the proportion of infants aged 8–12 months engaging in self-feeding is 55% in the UK [16], potentially making it the country with the highest prevalence. Conversely, in Asia, the practice of BLW is considerably less widespread [17,18,19]. This disparity can be attributed to caregivers exhibiting excessive caution and apprehension regarding potential accidents [20] arising from infants’ self-feeding, despite the infrequent occurrence of such issues [21,22].

The limited documentation on the practice of caregivers introducing complementary foods to infants in China has been noted [23]. Presently, it is prevalent for grandparents to assume the responsibility of infant care in China. Given their traditional upbringing of infants two to three decades ago, and social media being the main source of information on BLW [9], they may not be well-versed in contemporary infant care practices and may not adhere to current parenting trends. Furthermore, grandparents tend to exhibit excessive affection towards their grandchildren, potentially leading them to independently feed the infants. What is the present state of feeding practices during the introduction of complementary foods in infancy in China? Can it be improved through the provision of expert feeding guidance?

The objective of this study was to examine the prevalence of infants’ feeding patterns during the introduction of complementary foods and the factors influencing their self-feeding proportion in Beijing, China. Additionally, the study aimed to determine whether professional feeding guidance could enhance the practice of BLW during the introduction of complementary foods. The findings of this study will contribute to the body of evidence on improving BLW practices during the introduction of complementary foods, thereby facilitating better postnatal care in China.

## 2. Materials and Methods

### 2.1. Participants

The study was carried out between September 2022 and February 2023 at the Service Center of Integrated Early Childhood Development within Beijing Fengtai Maternal and Child Health Hospital, located in Beijing, China. The study was conducted by two seasoned pediatricians (G.S. and J.C., the former is an International Board Certificated Lactation Consultant (IBCLC, L-115089) who provides guidance over time through various sessions). Participants were recruited from Beijing, China, utilizing information disseminated on social media platforms. A total of 122 families, with infants aged 6–11 months who were healthy and born at full term, took part in the research. Prior to their involvement, all infants had already been introduced to complementary foods. During the study period, a total of 64 families were lost to follow-up as a result of the COVID-19 pandemic and the implementation of a national lockdown policy. To investigate the impact of professional feeding guidance on infants’ self-feeding proportion, 58 families were re-queried using questionnaires and their infants’ motor development was assessed on site one month later. No control group was employed for the researchers could not legitimately withhold a beneficial intervention from such a group.

The study was approved by the Ethical Committees of the Capital Institute of Pediatrics and Beijing Fengtai Maternal and Child Health Hospital (SHERLL2022046 and 2022FY-07, respectively). Participation in the study was voluntary and the infants’ parents signed a written informed consent prior to their participation.

### 2.2. Procedures

**Figure 1:**
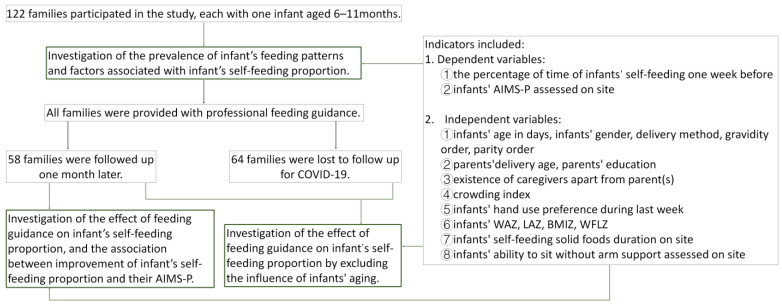
Flow chart of the study. Abbreviations: WAZ, weight-for-sex/age z-score; LAZ, length-for-sex/age z-score; BMIZ, BMI-for-sex/age z-score; WFLZ, weight-for-length for sex z-score; FAS, Family Affluence Scale; AIMS, Albert Infant Motor Scale; AIMS-P, Albert Infant Motor Scale for age percentile ranks.

The procedures consist of two parts (Figure 1).

#### 2.2.1. Investigating the Prevalence of Infants’ Feeding Patterns and Associated Factors

The primary dependent variable examined in this study was the proportion of self-feeding exhibited by infants during their meals at home. On the designated trial day, parents were requested to estimate the percentage of time spent on caregiver spoon feeding, infants independently reaching and feeding themselves solid foods, and infants autonomously spoon feeding themselves over the course of the previous week. The cumulative sum of these proportions equaled 100%.

The Albert Infant Motor Scale for age percentile ranks (AIMS-P) was utilized as the secondary dependent variable. The motor development level of each infant was evaluated by the pediatricians using the motor profiles mandated by the Albert Infant Motor Scale (AIMS), resulting in the acquisition of the AIMS-P for each infant. These scores were subsequently categorized into seven levels (<5th, 5th~10th, 10th~25th, 25th~50th, 50th~75th, 75th~90th, and >90th).

The independent variables included infants’ basic information (infant age in days, gender, Weight-for-sex/age z-score (WAZ), length-for-sex/age z-score (LAZ), BMI-for-sex/age z-score (BMIZ), and weight-for-length for sex z-score (WFLZ)), parental basic information (parental delivery age, delivery method, gravidity order, and, parity order), caring environment (parents’ education, families’ socioeconomic status parameters, and existence of caregivers apart from parent(s)), and infants’ development aspects (infants’ hand use preference during last week, infants’ self-feeding solid foods duration on site, and infants’ ability to sit without arm support). Parents were asked about most variables directly through questionnaires. The crowding index [24] was used to demonstrate the families’ socioeconomic status and was calculated by dividing the number of individuals, excluding infants, living in a dwelling per household, by the number of rooms in the house except for the kitchen, bathrooms, and toilets. Measurements for infants’ weight and length were standardized according to the WHO recommendations (WHO, 1995) and carried out by the pediatricians. The recumbent length of infants was measured on site to the nearest millimeter with portable devices equipped with height gauges. Their weight was assessed undressed to the nearest 100 g on Seca^®^ (Hamburg, Germany) electronic taring scales allowing for double weighing. The BMI was calculated as weight (kg) divided by height (m)^2^. WAZ, LAZ, BMIZ, and WFLZ were computed using the WHO multicenter growth reference standards’ [25] macro for R [26] (WHO, 2006).

**Figure 2:**
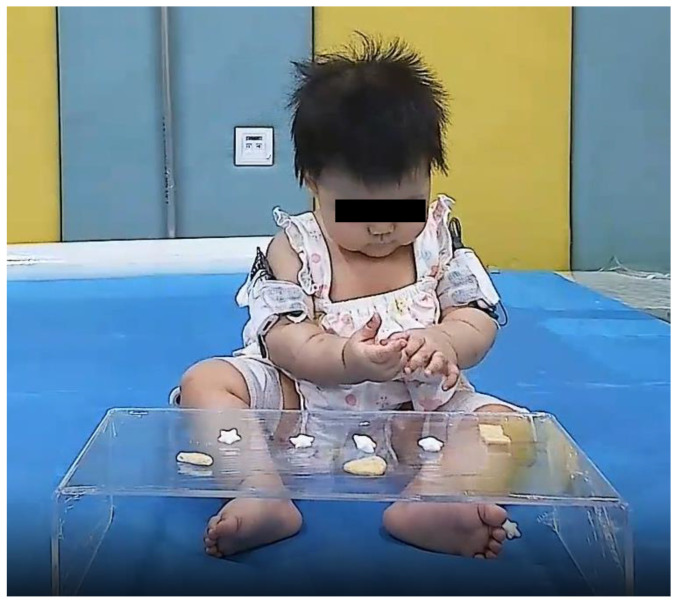
The infant ate solid foods on a small transparent table in front with a long sitting or ring sitting.

In order to obtain the infants’ self-feeding solid foods duration on site, prior to the trials, the parents were asked to prepare infantile solid foods according to China’s Nutrition guidelines of complementary feeding for infants and toddlers (WS/T 678—2020) [27] and the guidance from the UK’s National Health Service (NHS) [28] regarding complementary foods introduction. The course of the infants eating solid foods (Figure 2) was recorded by a camera in front of the infants. Infants’ ability to sit without arm support was assessed by the pediatricians through motor profiles required by AIMS.

#### 2.2.2. Investigating the Influence of Professional Feeding Guidance on the Infants’ Self-Feeding Proportion

All families were provided with comprehensive and personalized professional guidance on the introduction of complementary foods for infants, which was delivered by the pediatricians in accordance with China’s Nutrition guidelines for infants and toddlers (WS/T 678—2020) [27] and the guidance from the UK’s National Health Service (NHS) [28] on the introduction of complementary foods. The feeding guidance encompassed several key components, namely: (a) advocating for the advantages of infants engaging in self-feeding benefits of infants engaging in self-feeding, (b) providing information regarding the selection of nutritionally balanced foods (particularly those high in iron content, such as red meat, animal liver, and iron-rich vegetables), (c) instructing on the techniques for shaping food into a size comparable to an adult’s middle finger and ensuring an appropriate texture suitable for infants’ grasping and chewing capabilities, (d) emphasizing the significance of developing stable sitting skills for successful self-feeding, and (e) addressing emergency situations, including the occurrence of vomiting reflex, food expulsion from the mouth, gagging, and choking, along with their respective management strategies.

The intervention remained consistent across all participating families. Part (b) and part (c) of the feeding guidance were shared with families one day prior to the official trial day. Caregivers were instructed to prepare solid foods suitable for the infant’s age and developmental stage. They were also encouraged to document the food preparation process through videos or pictures, which were shared with the researchers via WeChat. This process aimed to ensure that caregivers had a comprehensive understanding of the feeding guidance. The prepared foods were then brought to the evaluation site. During the assessment, as the infants independently consumed solid foods, the researchers addressed issues outlined in parts a), d), and e) of the feeding guidance with the caregivers. In cases where infants displayed symptoms such as vomiting reflex, food expulsion, gagging, or choking, the researchers provided on-site demonstrations of appropriate handling techniques.

Due to the pandemic and the national lockdown policy of COVID-19 during the study period in China, 64 families were lost to follow-up. A total of 58 families were re-queried through questionnaires and assessed infants’ motor development on site one month later for the purpose of investigating the influence of professional feeding guidance on the infants’ self-feeding proportion.

To mitigate the potential impact of infants’ developmental progression, a total of 64 families were examined prior to receiving feeding guidance, while 58 families were analyzed after receiving such guidance. The families were further divided into two groups based on the age range of their infants, specifically 6–8 months and 9–11 months. The comparison was made between these groups in terms of the provision of feeding guidance. Additionally, this study also analyzed the changes in infants’ self-feeding proportion and AIMS-P scores for the aforementioned 58 infants before and after receiving feeding guidance.

### 2.3. Statistical Analysis

The description of the characteristics of all the families was conducted first. Continuous variables were described as mean (SD) (or median [IQR] for non-normal data). Categorical variables were described as numbers (%). Shapiro–Wilk tests were used to test the normality of the continuous variables. A generalized linear model was used to identify the factors associated with the proportion of infants’ self-feeding before participation. The dependent variable of the model was the proportion of infants’ self-feeding; infants’ age, gender, WAZ, maternal delivery age, paternal delivery age, delivery style, gravidity order, parity order, maternal education level, paternal education level, caregivers’ type, crowding index, hand use preference, self-feeding solid foods duration in the observation, and the ability to sit without arm support were independent variables. LAZ, BMIZ, and WFLZ were not included for their collinearity with WAZ. Only sitting skills, not infants’ AIMS-P, were included because of their collinearity too. The stepwise regression method and calculation of standardized regression coefficients, which could address dimensional issues, were used to obtain the optimal generalized linear regression model.

Paired Wilcoxon rank-sum test was carried out to compare the infants’ self-feeding proportion of 58 families before and after professional feeding guidance. Wilcoxon rank-sum test was applied to compare the infants’ self-feeding proportion between families without and with feeding guidance by excluding the influence of aging. χ^2^ test was used to explore the association between improvement in infants’ self-feeding proportion and infants’ AIMS-P among 58 infants. *p*-values < 0.05 were considered statistically significant. Statistical analysis was performed using R 4.1.0.

## 3. Results

### 3.1. Basic Characteristics of the Families

A total of 122 infants were introduced to complementary foods prior to their participation, as indicated in Table 1. The majority of infants were fed by their caregivers, with only a 10% [0%, 40%] proportion of infants engaging in self-feeding during each meal. The self-feeding rate for infants aged 6–8 months (*n* = 74) was exceptionally low at 0% [0%, 30%], while it was 20% [10%, 50%] for infants aged 9–11 months. It is worth mentioning that all anthropometric indicators (WAZ, LAZ, BMIZ, and WFLZ) were above −3, and all AIMS-Ps were equal to or greater than the fifth percentile.

### 3.2. The Factors Associated with the Proportion of Infants’ Self-Feeding

The findings from Table 2 indicate a significant positive association between infants’ self-feeding proportion and their age (95%CI 0.01 to 0.19, *p* = 0.028). To address the issue of dimension inconsistency, a stepwise management approach was employed, and standardized regression coefficients were computed. This process led to the derivation of the final optimal generalized linear regression equation: infants’ self-feeding proportion = 0.200 (infant age) − 0.128 (caregivers other than parent(s)).

### 3.3. The Effect of Professional Feeding Guidance on the Proportion of Infants’ Self-Feeding

**Figure 3:**
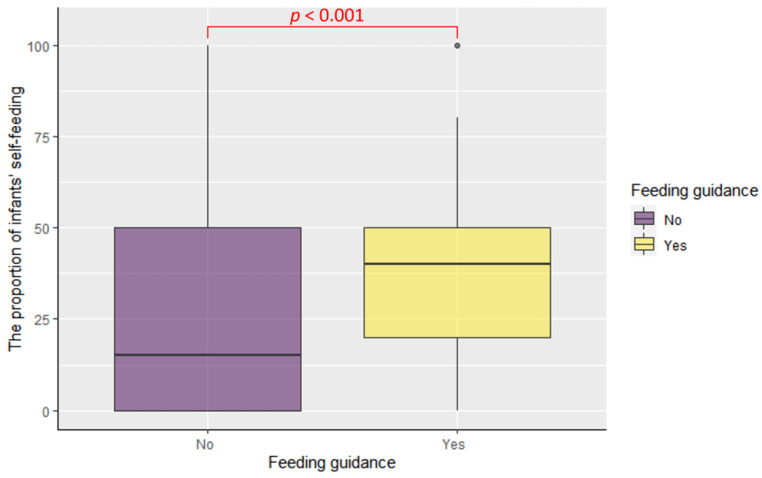
The comparison of proportion of infants’ self-feeding of 58 families between before and one month after professional feeding guidance.

The study examined a cohort of 58 families and found a significant increase in the proportion of infants engaging in self-feeding during the introduction of complementary foods. This increase was observed from 15% [0%, 50%], prior to receiving professional feeding guidance, to 40% [20%, 50%], one month after receiving professional feeding guidance (*p* < 0.001, Figure 3).

### 3.4. The Effect of Professional Feeding Guidance on the Infants’ Self-Feeding Proportion by Excluding the Influence of Infants’ Aging

**Figure 4:**
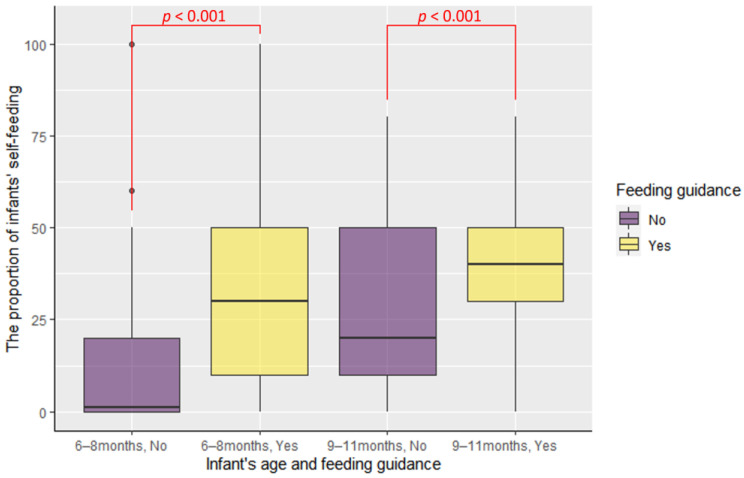
The comparison of infants’ self-feeding proportion between families which did not accept the feeding guidance and received feeding guidance.

To control for the potential influence of infants’ aging, the study analyzed a total of 64 families prior to receiving feeding guidance and 58 families after receiving feeding guidance. Within these groups, separate comparisons were made between families with infants aged 6–8 months and families with infants aged 9–11 months, based on the presence or absence of feeding guidance. The results showed a significant increase in infants’ self-feeding proportion following professional feeding guidance (from 1% [0%, 20%] to 30% [10%, 50%], *p* < 0.001 for 6–8 months old infants; from 20% [10%, 50%] to 40% [30%, 50%], *p* < 0.001 for 9–11 months old infants; see Figure 4).

The χ^2^ test conducted on the aforementioned 58 families revealed that, following professional feeding guidance, 38 infants (65.52%) demonstrated an increase in self-feeding behavior compared to their previous state. However, only 14 infants exhibited an increase in their AIMS-P scores, indicating a lack of significant association between these two variables (refer to Table 3).

## 4. Discussion

This study represents the initial investigation into the prevalence of infants’ self-feeding complementary foods in China, as well as the exploration of factors associated with this proportion. The research methodology involved combining on-site assessments of infants’ self-feeding courses and motor development in China. The findings indicate an alarmingly low proportion of infants self-feeding complementary foods in China. However, it was observed that, through the implementation of professional feeding guidance, a significant increase in the proportion of infants’ self-feeding can be achieved. This finding addressed the disparity in the prevalence of self-feeding complementary foods among Chinese infants and put forth a corresponding solution, thus holding substantial importance for enhancing infant care.

In our study, the prevalence of infants engaging in self-feeding was found to be only 10% [0%, 40%]. Similarly, Justine Briaux et al. [29] reported that the proportion of infants aged 6–11 months who were able to eat without assistance during meals was only 10.78%. The optimal standardized generalized linear regression equation, which was derived from our study, indicated a positive association between infants’ age and their self-feeding proportion. Furthermore, a study conducted in Spain also demonstrated an increase in the percentage of women who reported using baby-led weaning as their infants’ age advanced [30]. It is evident that, as infants develop, their sitting posture becomes increasingly stable, their ability to accurately reach and transport food to their mouths improves, and their chewing and swallowing become safer, resulting in a transition from consuming porridge or purées with a spoon to consuming food in pieces, strips, or sticks [31,32]. This shift in eating habits leads to a more efficient and less anxiety-inducing eating experience [33]. Consequently, providing infants with increased opportunities to independently feed themselves did not demonstrate any correlation with the development of sitting without arm support. This lack of association may be attributed to the fact that infants were seated in a secure high chair, irrespective of their attainment of independent sitting ability. As a result, their sitting posture remained stable, enabling them to effectively handle solid foods with mature reaching and grasping abilities from the age of 6 months [34].

The prevalence of infants engaging in self-feeding appears to diminish when entrusted to caregivers other than their parent(s). This phenomenon may be attributed to the inclusion of grandparents as caregivers in China, who tend to exhibit a high level of affection towards their grandchildren. Transgenerational experience plays an important role in complementary feeding in infants [35]. These grandparents, having raised infants two to three decades ago, adhere to traditional methods of infant care and may not be well-versed in contemporary parenting approaches. These caregivers demonstrated a high level of caution and concern regarding potential accidents [20] and the nutritional implications associated with infants engaging in self-feeding [22], despite the infrequency of such issues [21,22]. Consequently, the perspectives of caregivers may influence the extent to which infants engage in self-feeding. Therefore, the effective dissemination of accurate information regarding the introduction of complementary foods, particularly self-feeding practices among infants, is of utmost importance.

Following the provision of professional guidance on the introduction and management of complementary foods in emergency situations, such as vomiting reflex, food expulsion from the mouth, gagging, and choking during feeding, there was a significant increase in the proportion of infants engaging in self-feeding after one month. Important to acknowledge is the constraint posed by the limited sample size and high attrition rate in our study, primarily attributed to the non-random impact of the COVID-19 pandemic and subsequent lockdown policies. Notwithstanding these challenges, the conclusions of the study remain unaffected. With the evolution of China’s COVID-19 policies, there exists an opportunity for conducting further studies with similar parameters. Nevertheless, the advantage of our study was the exclusion of the influence of infants’ aging. Notably, the proportion of infants engaging in self-feeding remained significantly higher after receiving professional feeding guidance, particularly among infants aged 6–8 months. The findings of this study highlight the significant impact of professional feeding guidance on the self-feeding practices of infants. It suggests that such guidance can effectively enhance infants’ self-feeding abilities, regardless of their age. Notably, in Italy, there has been a notable shift in feeding practices from traditional spoon feeding to Baby-Led Weaning (BLW) styles, as evidenced by a nationwide increase in the proportion of primary care providers (PCPs) endorsing BLW between 2015 and 2017 [36]. This shift underscores the feasibility and value of implementing professional feeding guidance. It is imperative to actively promote pertinent guidelines [27,28] and ensure the comprehensive training of a substantial number of healthcare providers specializing in children’s healthcare, with the aim of disseminating relevant knowledge widely. While our study was unable to discern the specific extent to which the observed rise in self-feeding post-professional guidance could be attributed to the guidance itself as opposed to the natural developmental progression linked with age, a more meticulously designed study may offer a quantitative resolution to this issue. Additionally, the potential existence of a Promotion–Prevalence Paradox [37], similar to the one observed in the promotion of breastfeeding, requires exploration in a larger sample, necessitating the comprehensive analysis of factors such as race, socioeconomic status, and others in the future.

The study’s limitation was the short interval between two participations. Consequently, this factor may explain the lack of a significant association between the improvement in infants’ self-feeding proportion and AIMS-P, despite previous studies confirming such a relationship [10,11,12]. Therefore, it is recommended to conduct a more extensive longitudinal study to further investigate the correlation between these variables. Furthermore, the survey did not encompass an examination of parents and caregivers’ understanding of the introduction of complementary foods for infants. Given that this understanding [38] could potentially mediate the relationship between the low proportion of infants engaging in self-feeding and the involvement of caregivers other than the parent(s), it is imperative that future research investigates their knowledge in order to develop comprehensive and tailored professional feeding guidance.

Our study, conducted exclusively in Beijing, China, limits the generalizability of the findings to other regions within the country due to potential variations in cultural, economic, and social factors. Moreover, the research was conducted during the peak of the COVID-19 lockdown in China, with recruitment concluding upon reaching 122 families, thus preventing a comprehensive representation of Beijing, let alone the entirety of China. However, given Beijing’s pivotal role in influencing national policymaking, our study’s findings, along with the devised content and implementation methods for feeding guidance, retain some relevance in informing the introduction of complementary foods for infants nationwide. Future endeavors may entail a multi-center approach to mitigate potential biases.

## 5. Conclusions

The prevalence of infants engaging in self-feeding during the introduction of complementary foods in Beijing, China was found to be remarkably low. However, the provision of our professional feeding guidance pertaining to the introduction of complementary foods has the potential to substantially enhance this proportion. Consequently, our study offers valuable insights that can inform improved postnatal care practices and provide a template for feeding guidance within the domain of infants’ complementary foods introduction.

## Figures and Tables

**Table 1:** Basic characteristics of all the families participating in the study.

Characteristic	*n* = 122
Proportion of infants’ self-feeding (median [IQR])	10% [0%, 40%]
6–8 months (*n* = 74)	0% [0%, 30%]
9–11 months (*n* = 48)	20% [10%, 50%]
Infant age in days (median [IQR])	246.00 [205.75, 295.75]
Boys—no. (%)	69 (56.6)
WAZ (mean (SD))	0.55 (0.95)
LAZ (mean (SD))	0.52 (1.10)
BMIZ (mean (SD))	0.35 (0.92)
WFLZ (mean (SD))	0.45 (0.91)
Maternal delivery age (median [IQR])	31.00 [29.00, 34.00]
Paternal delivery age (median [IQR])	32.00 [29.25, 36.00]
Vaginal delivery—no. (%)	82 (67.2)
Gravidity ordered 1st—no. (%)	92 (75.4)
Parity ordered 1st—no. (%)	104 (85.2)
Mother with less than master’s degree—no. (%)	71 (58.2)
Father with less than master’s degree—no. (%)	80 (65.6)
Existence of caregivers apart from parent(s)—no. (%)	112 (91.8)
Crowding index (median [IQR])	1.67 [1.33, 2.00]
Hand use preference	
No preference	75 (61.5)
Left preference	13 (10.7)
Right preference	34 (27.9)
Self-feeding solid foods duration in the observation (median [IQR])	10.00 [8.00, 15.75]
The ability to sit without arm support—no. (%)	97 (79.5)
AIMS-P	
<5th	0 (0)
5th~10th	7 (5.7)
10th~25th	11 (9.0)
25th~50th	11 (9.0)
50th~75th	31 (25.4)
75th~90th	28 (23.0)
>90th	34 (27.9)

Abbreviations: WAZ, weight-for-sex/age z-score; LAZ, length-for-sex/age z-score; BMIZ, BMI-for-sex/age z-score; WFLZ, weight-for-length for sex z-score; AIMS, Albert Infant Motor Scale; AIMS-P, Albert Infant Motor Scale for age percentile ranks.

**Table 2:** Generalized linear model regarding infants’ self-feeding proportion.

Indicators	β	Standardized β	95%CI	*p*
(Intercept)	92.783			<0.001 **
Infants’ age in days	0.098	0.200	0.01, 0.19	0.028 *
Caregivers apart from parent(s)	−11.457	−0.128	−27.46, 4.55	0.159

* *p <* 0.05, ** *p <* 0.001.

**Table 3:** The association between improvement in infants’ self-feeding and motor development after professional feeding guidance.

	No AIMS-P Increase	AIMS-P Increase	χ^2^	*p*
No improvement in infants’ self-feeding	13	7	1.1657	0.2803
Improvement in infants’ self-feeding	31	7		

Abbreviations: AIMS-P, albert infant motor scale for age percentile ranks.

## Data Availability

Not applicable.
